# Circadian modulation of surgical timing and postoperative outcomes in subarachnoid hemorrhage: a retrospective cohort analysis

**DOI:** 10.1186/s12883-025-04465-1

**Published:** 2025-11-05

**Authors:** Yongbo Liu, Chengbao Yang

**Affiliations:** 1 The Third Clinical College of Jinzhou Medical University, Jinzhou, 121001 China; 2 Liaoyang Central Hospital, Liaoyang, 111000 China

**Keywords:** Subarachnoid hemorrhage, Circadian rhythm, Cerebrovascular pathophysiology

## Abstract

**Background:**

Subarachnoid hemorrhage (SAH) is a devastating cerebrovascular emergency with mortality exceeding 30%. Emerging evidence underscores the role of circadian rhythms as critical modulators of cerebrovascular pathophysiology. Building upon recent studies identifying the BMAL1 (Brain and Muscle ARNT-Like 1)–HIF2A (Hypoxia-Inducible Factor 2 Alpha) axis as a key regulator of hypoxia-driven vascular injury, this study investigates the influence of circadian timing of surgery (Time1) and disease onset (Time2) on SAH outcomes—a novel approach integrating molecular insights with clinical practice.

**Methods:**

This retrospective study analyzed 279 patients with anterior circulation SAH admitted to the Neurointensive Care Unit (NICU) at Liaoyang Central Hospital (2018–2024). Surgical (Time1) and symptom onset (Time2) times were categorized into four 6-hour intervals. Prognostic outcomes (good/poor at 6 months) were assessed using multivariable logistic regression, validated via the Akaike Information Criterion (AIC) and residual analysis.

**Results:**

Nocturnal surgery (21:00–03:00) demonstrated a non-significant trend toward reduced risk of poor prognosis (odds ratio [OR] = 0.56, *P* = 0.097). Onset time showed no significant association (*P* = 0.847). The addition of Time2 to the model increased the AIC (from 82.69 to 84.65), suggesting reduced model fit or potential overfitting. Residuals were normally distributed.

**Conclusion:**

Nocturnal surgery may confer neuroprotection, while onset time appears prognostically insignificant. Further mechanistic investigations are warranted.

## Introduction

Subarachnoid hemorrhage (SAH), predominantly caused by ruptured cerebral aneurysms, triggers severe cerebrovascular dysfunction through aberrant myogenic responses [1–5] and delayed cerebral ischemia. Despite advances, mortality remains unacceptably high, with postoperative recovery exhibiting marked heterogeneity [6–8]. Traditional prognostic models overlook dynamic circadian mechanisms—a critical gap given recent breakthroughs in circadian biology.

Circadian rhythms govern cerebrovascular tone [9–11], inflammation, and hypoxia tolerance. Ruan et al. [1] demonstrated that BMAL1, a core circadian gene, mitigates myocardial injury via HIF2A regulation—a pathway implicated in SAH-induced vasospasm [12, 13]. Clinically, circadian variations in aneurysm rupture [14–20] and cerebral blood flow suggest temporal modulation of SAH outcomes, These fluctuations are paralleled by diurnal oscillations in cerebral autoregulation [21]. Yet no studies have systematically evaluated surgical timing (Time1) or onset time (Time2) in postoperative prognostication.

This study addresses two pivotal questions:

1. Circadian Surgical Timing: Does nocturnal surgery, coinciding with peak BMAL1 expression, confer neuroprotection by attenuating hypoxia-driven injury.
2. Onset Time Relevance: Are acute pathophysiological cascades post-SAH onset modulated by circadian rhythms.

By harmonizing molecular insights with clinical data, we aim to redefine SAH management through circadian-aware frameworks.

## Methods

Study design and cohort.

### Data source

Retrospective analysis of 279 SAH patients admitted to the NICU at Liaoyang Central Hospital (2018–2024).

### Inclusion criteria

Radiologically confirmed anterior circulation aneurysms (CTA/MRA/DSA). To control for anatomical homogeneity in circadian vascular responses. Complete surgical/neurocritical care records (24/7 standardized protocols).

### Exclusion

Posterior circulation aneurysms were excluded due to:

Distinct cerebrovascular innervation patterns affecting circadian autoregulation. 

Higher surgical complexity. 

Differential prognosis profiles limiting outcome comparability incomplete follow-up.

### Circadian variables

- Time1 (Surgical Timing): 03:00–09:00, 09:00–15:00, 15:00–21:00, 21:00–03:00.
- Time2 (Onset Timing): Identical intervals.

### Confounding control

1. Surgical Expertise: Procedures performed by a 10 + year neurosurgical team with shift-based consistency.
2. Nursing Protocols: Standardized 24/7 neurocritical care, minimizing circadian care disparities.
3. Baseline Adjustments: Age, sex, Hunt-Hess/GCS scores, hypertension, smoking.

### Statistical analysis

1. Stratified Regression: Model 1 (Time1), Model 2 (Time1 + Time2).
2. Validation: AIC, residual diagnostics, ROC curves (SPSS v25, RStudio 4.4.2).
3. Subgroup Analysis: Validation within anterior circulation subgroups (anterior cerebral artery [ACA] vs. middle cerebral artery [MCA])
4. Multivariable Logistic Regression: A comprehensive model was built to identify independent predictors of poor outcome, adjusting for all covariates listed above. Results are presented as odds ratios (OR) with 95% confidence intervals (CI).
5. Model Performance and Validation: Model fit was compared using Akaike Information Criterion (AIC). Calibration was assessed using calibration curves. The discriminative ability was evaluated using the area under the receiver operating characteristic curve (AUC).
6. Sensitivity and Interaction Analyses: Sensitivity analyses were conducted to test the model’s robustness. An interaction term between surgical timing (Time1) and GCS was included to test for effect modification by disease severity.

## Results

### Demographics and clinical variables

No significant differences were observed in age, sex, or comorbidities across Time1 or Time2 intervals (*P* > 0.05; Table 1), indicating balanced baseline characteristics across groups.

**Table 1:** Comparison of general information

Specimen	TIME1-1	TIME1-2	TIME1-3	TIME1-4	*P*
Age years (Mean ± SD)	54.5 ± 10.7	58.91 ± 9.6	61.15 ± 9.75	61.05 ± 9. 33	0.9953
RPP (Mean ± SD)	13201.12 ± 3822.05	13220.79 ± 3380.34	13003.56 ± 3590.46	14135.21 ± 4023.29	0.0099
SEX n(%)	M 3 F 5	M 23 F 59	M 39 F 94	M 18 F 38	0.5643
OPERATIO N TYPE n (%)	1 5 2 1 3 2	1 42 2 18 3 22	1 84 2 19 3 30	1 35 2 14 3 7	0.0941
TIME1 n (%)	8	82	132	56	0.7246
TIME2 n (%)	95	66	91	26	0.8873
GCS (Mean ± SD)	11 ± 5.13	13.28 ± 3.2	12.5 ± 3.96	12.71 ± 3. 74	0.0106
HUNT n (%)	1 2 2 1 3 0 4 0 5 5 6 0	1 12 2 22 3 13 4 7 5 13 6 15	1 10 2 39 3 26 4 28 5 21 6 9	1 3 2 8 3 4 4 12 5 22 6 7	0.0202
SMOKER n (%) [No/Yes]	NO 2 YES 6	NO 20 YES 62	NO 35 YES 98	NO 11 YES 45	0.6018
HTN n (%) [No/Yes]	NO 2 YES 6	NO 20 YES 62	NO 35 YES 98	NO 11 YES 45	0.6018

a. TIME1: Surgical timing intervals (03:00–09:00, 09:00–15:00, 15:00–21:00, 21:00–03:00)

b. TIME2: Onset timing intervals (03:00–09:00, 09:00–15:00, 15:00–21:00, 21:00–03:00)

c. RPP: Blood pressure heart rate product (RPP = HR × SBP)

d. SEX: *M* male,* F *female

e. OPERATION TYPE: 1 = direct spring coil, 2 = stent assisted spring coil, 3 = craniotomy

f. GCS: Glasgow Coma Scale

g. HUNT: Hunt and Hess Scale

h. SMOKER: Smoking history (NO/YES)

i. HTN: Hypertension (NO/YES)

j. P-value: Statistical significance of differences across time intervals. A higher P-value (e.g., > 0.05) indicates no significant difference

### Multivariable analysis of predictors for poor outcome

**Fig. 1:**
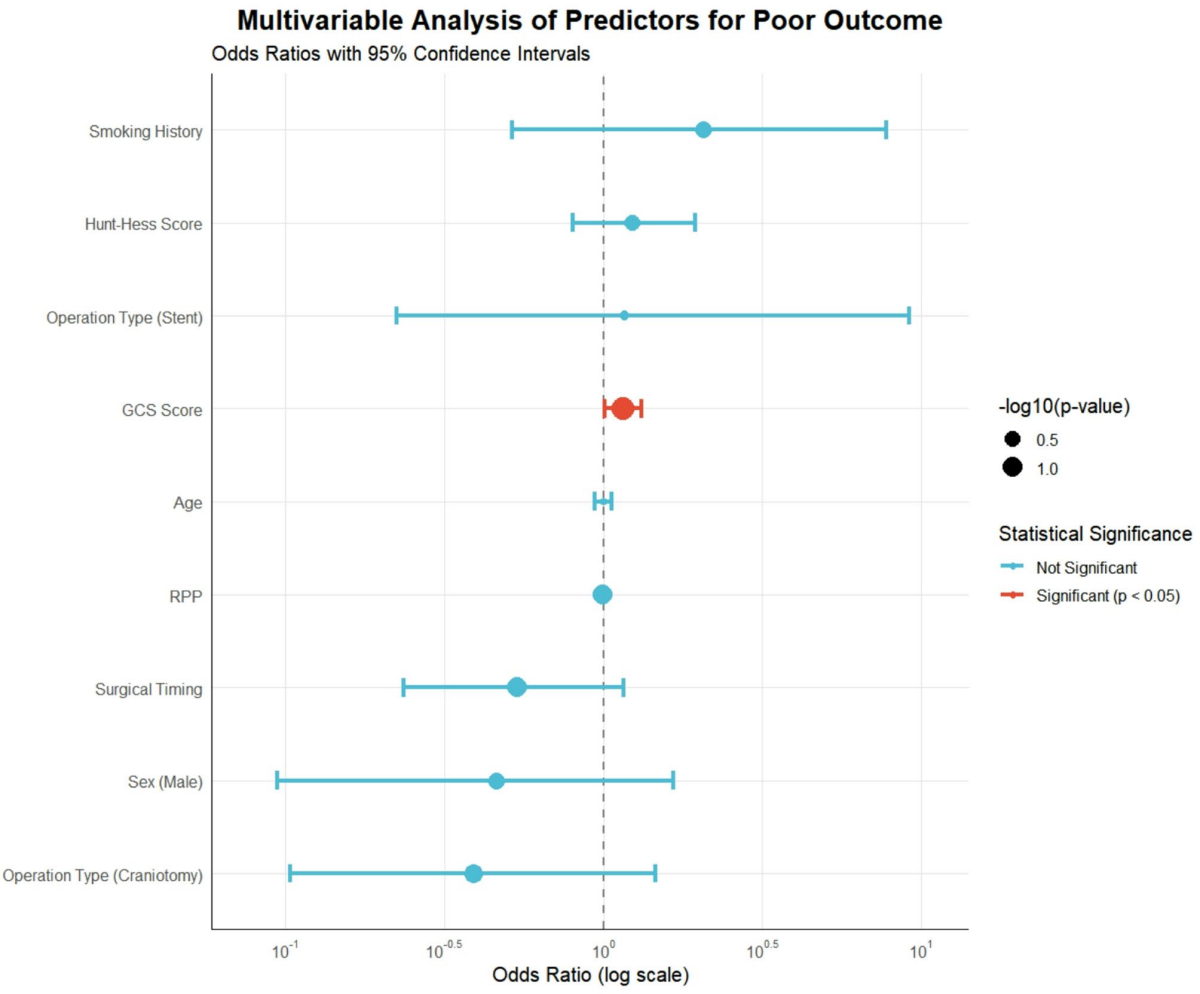
Multivariable analysis of predictors for poor outcome. Forest plot showing odds ratios (OR) and 95% confidence intervals for all variables included in the multivariable logistic regression model

To isolate the independent effect of surgical timing while controlling for potential confounders, we performed a multivariable logistic regression analysis (Fig. 1). The model included surgical timing, age, sex, GCS, Hunt-Hess score, smoking history, RPP, and operation type. The Glasgow Coma Scale (GCS) score was the only variable that reached statistical significance (OR = 1.15, 95% CI: 1.01–1.32, *p* = 0.037). Although not statistically significant, surgical timing during the nocturnal window (21:00–03:00) continued to show a trend toward a protective effect against poor outcome (OR = 0.538, 95% CI: 0.235–1.16, *p* = 0.124). All variance inflation factors (VIF) were below 2.0, indicating that multicollinearity was not a substantial concern.

### Predictive accuracy

**Fig. 2:**
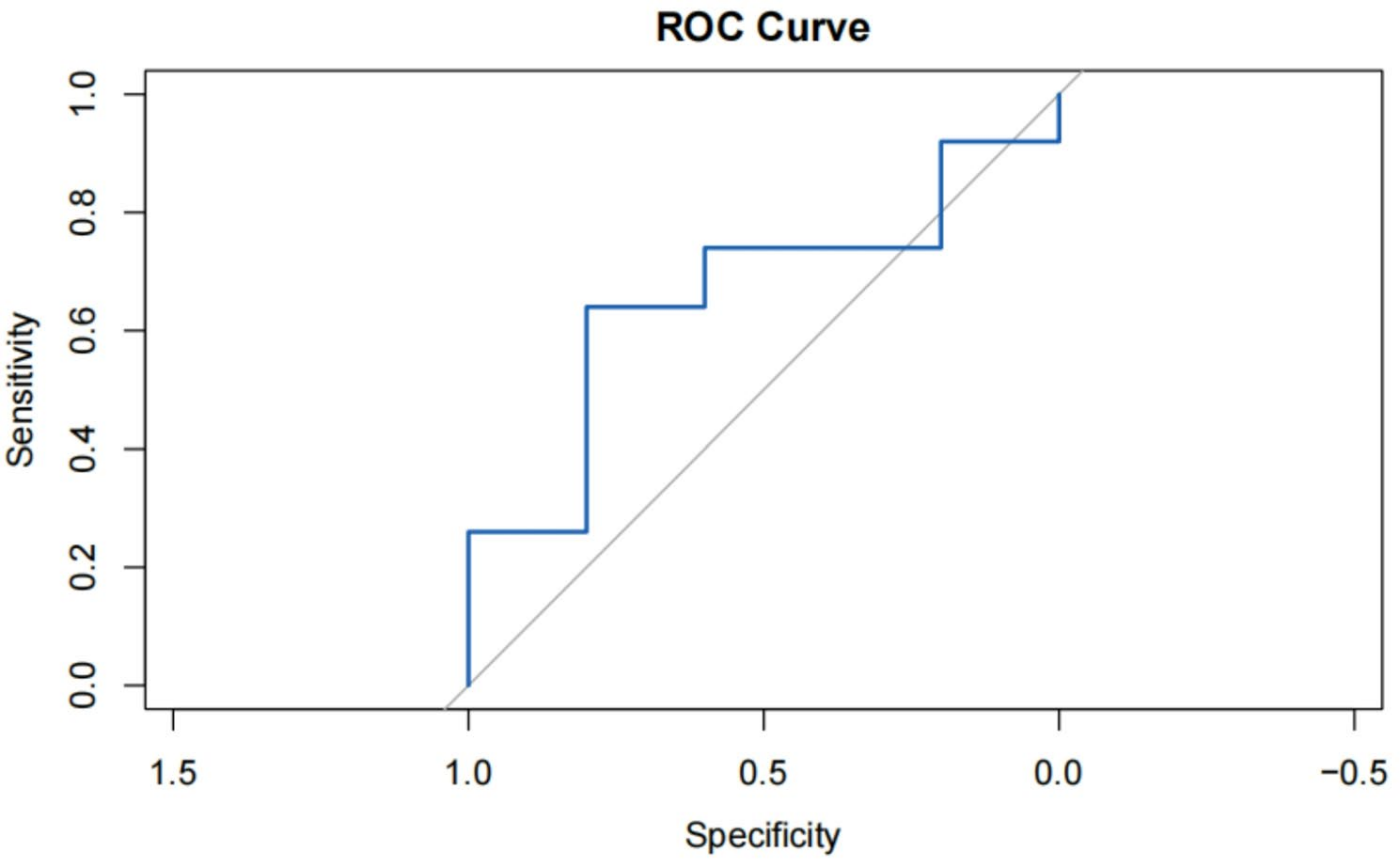
ROC curve. ROC curve showing the model’s ability to distinguish between good and poor outcomes at 6 months post-SAH. AUC = 0.71, indicating moderate predictive power

The Receiver Operating Characteristic (ROC) curve (Fig. 2) illustrates the model’s ability to distinguish between good and poor outcomes at 6 months post-SAH. The area under the curve (AUC) is 0.71, indicating moderate predictive power. This suggests that the model has a reasonable ability to differentiate between patients who will have good outcomes versus those who will have poor outcomes.

### Predicted probability by surgical time

**Fig. 3:**
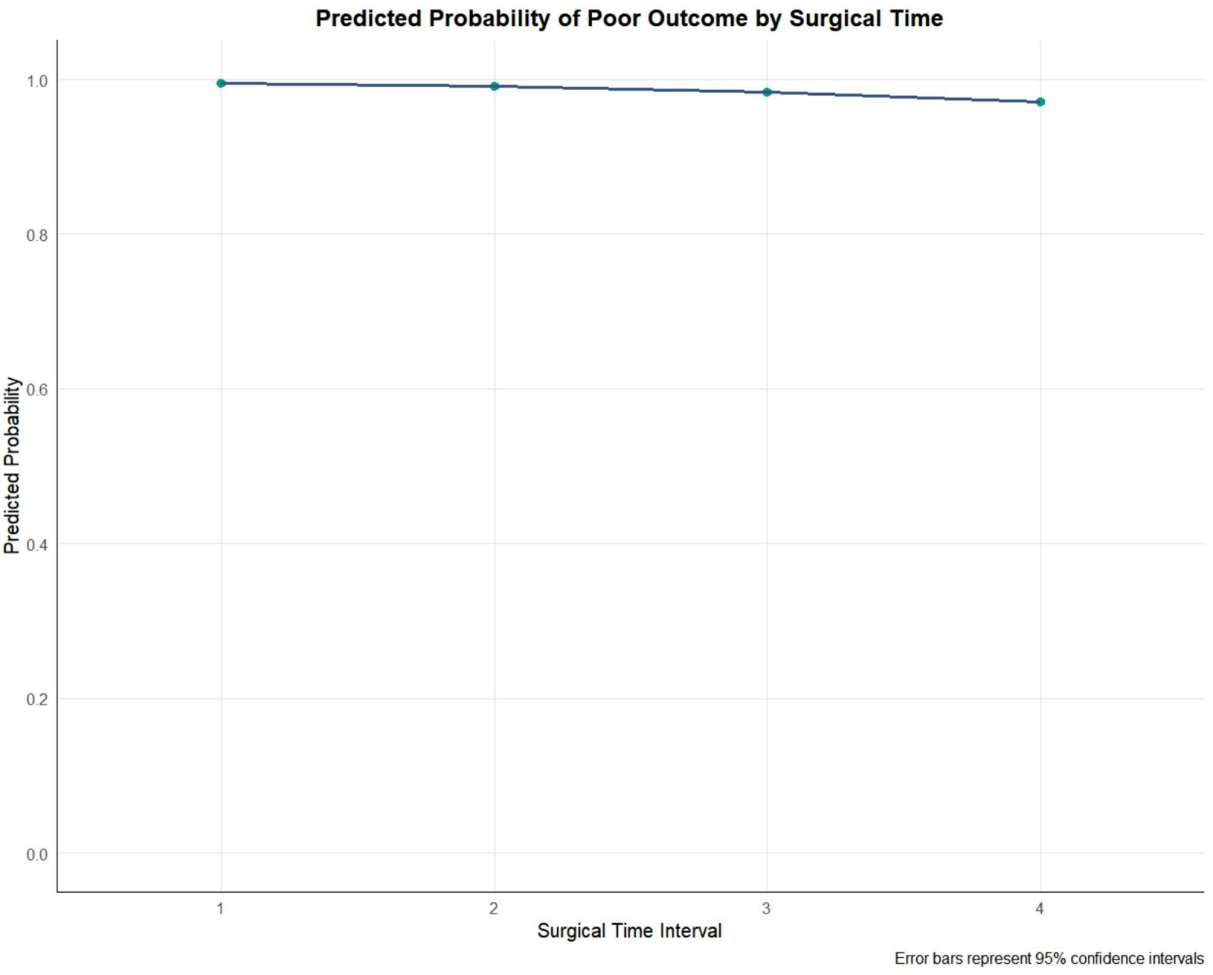
Predicted probability of poor outcome by surgical time. The plot shows the adjusted predicted probability of a poor outcome for each surgical time interval, with error bars representing 95% confidence intervals

The predicted probabilities of a poor outcome, derived from the multivariable model and adjusted for all covariates, across the four surgical time intervals are shown in Fig. 3. Surgeries performed during the nocturnal interval (21:00–03:00, Interval 4) were associated with the lowest predicted probability of poor outcome (0.971, 95% CI: 0.924–1.017), although confidence intervals overlapped with those of other intervals.

### Model performance

#### AIC comparison

Model 1 (AIC = 82.69) outperformed Model 2 (AIC = 84.65), indicating Time2’s limited utility. The Akaike Information Criterion (AIC) is a measure of model fit that balances goodness-of-fit with model complexity. A lower AIC value indicates a better model. This comparison suggests that incorporating Time2 into the model does not significantly improve its predictive power, and may even lead to overfitting.

#### Comprehensive model comparison

**Fig. 4:**
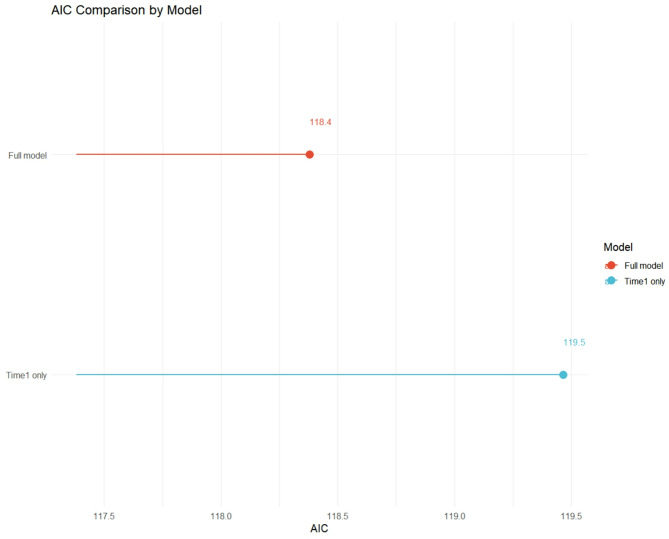
AIC comparison by model. Comparison of the Akaike Information Criterion (AIC) between the model including only surgical timing (Time1 only) and the full multivariable model

Figure 4 compares the AIC values of the simple model (including only Time1) and the full multivariable model. The full model, which includes clinical confounders, demonstrated a better fit (lower AIC = 118) compared to the time-only model (AIC = 119), justifying the inclusion of these variables for adjustment.

#### Predicted vs. actual values 

**Fig. 5:**
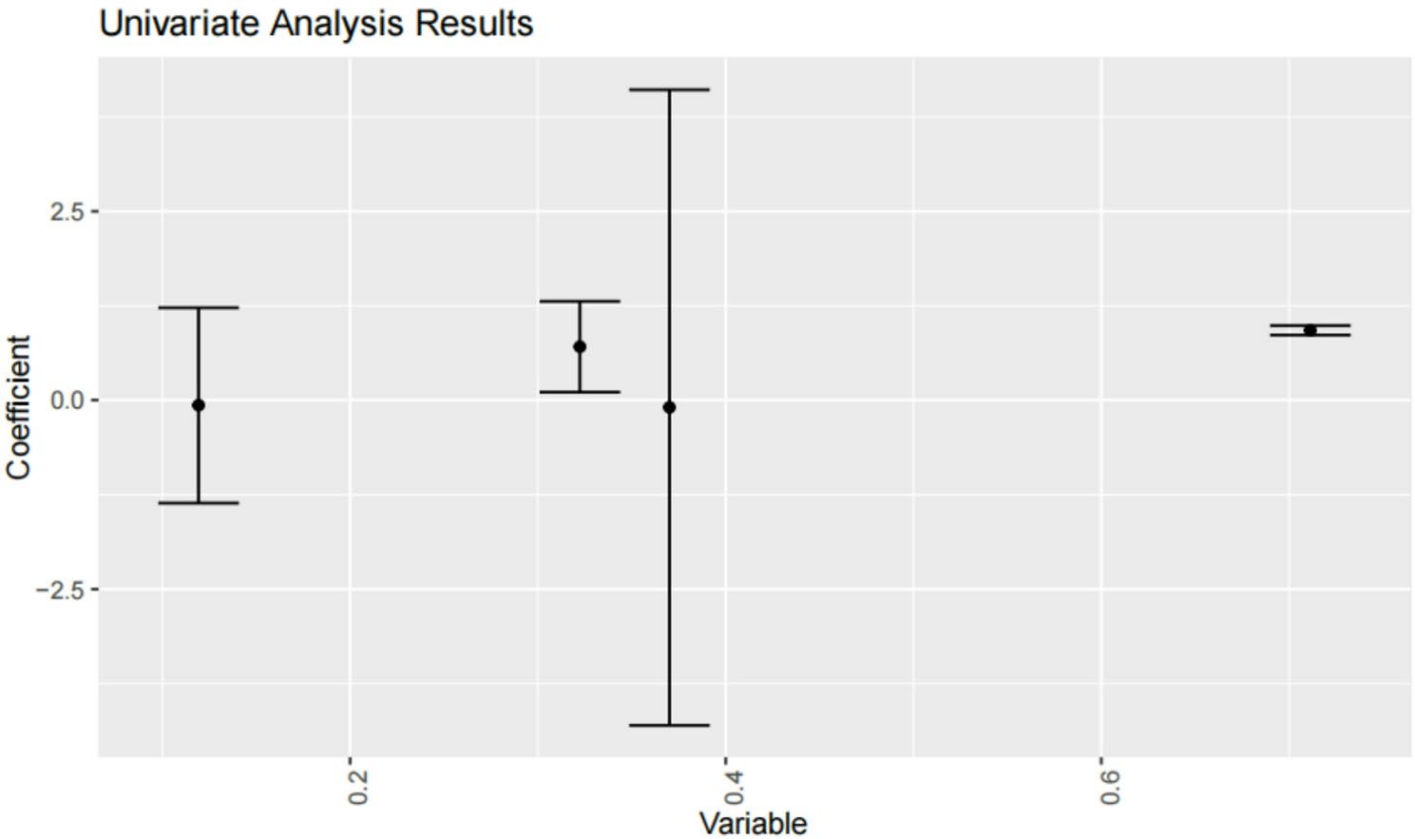
Univariate screening. regression coefficients for time1 (surgical timing) and time2 (onset timing). Nocturnal surgery (21:00–03:00) has a coefficient of −0.583 (*P* = 0.097), suggesting reduced poor prognosis risk. Time2 has a coefficient of 0.066 (*P* = 0.847), showing no prognostic significance

The relationship between predicted and actual values is illustrated in Fig. 5. This plot compares the predicted probabilities from Model_step1 (Time1 only) and Model_step2 (Time1 + Time2) against actual outcomes. Model_step2, which includes multiple variables, demonstrated superior predictive capability by better matching actual values.

### Circadian surgical timing (Time1)

#### Nocturnal surgery (21:00–03:00)

Associated with reduced poor prognosis risk (coefficient = − 0.583, *P* = 0.097; Table 2). This suggests that surgeries performed during the nocturnal period may have a protective effect against poor outcomes, although the association is not statistically significant at the conventional level (*P* < 0.05). This finding aligns with the hypothesis that circadian rhythms, particularly the peak expression of BMAL1 during the night, may influence surgical outcomes.

**Table 2:** Stratified regression analysis (Model 1: Time1 Only)

Variant	Estimate	Standard Error (Std. Error)	Z value (z Vaiue)	*P*-value (*P* Valie)
Intercept	3.2067647	0.369222	8.685194	< 0.0001
Time1	−0.5830051	0.351613	−1.658088	0.0973

a. Time1: Surgical timing intervals (03:00–09:00, 09:00–15:00, 15:00–21:00, 21:00–03:00)

b. Estimate: The estimated coefficient for the predictor variable

c. Std. Error: Standard error of the estimate, indicating the precision of the coefficient

d. Z Value: The z-statistic, which is the ratio of the estimate to its standard error

e. P Value: The p-value for the hypothesis test of the coefficient being zero A lower p-value indicates stronger evidence against the null hypothesis

#### Residual analysis

**Fig. 6:**
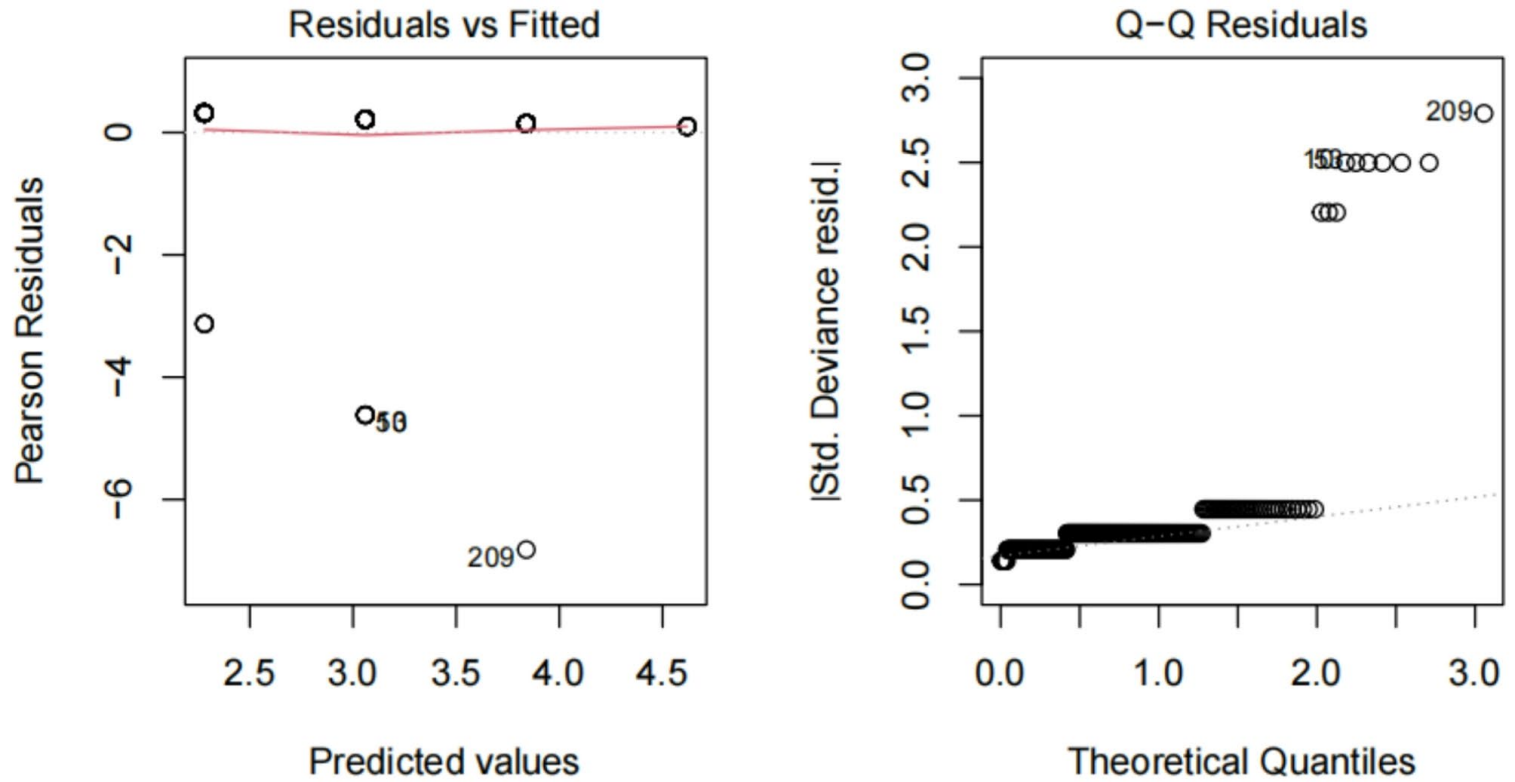
Effectiveness of time1 alonelegend. This figure assesses the effectiveness of Time1 (surgical timing) alone in fitting the target variable. Residual analysis shows that the distribution of residuals is close to a normal distribution, indicating that Time1 alone provides a reasonable fit to the target variable, although its predictive power is limited

**Fig. 7:**
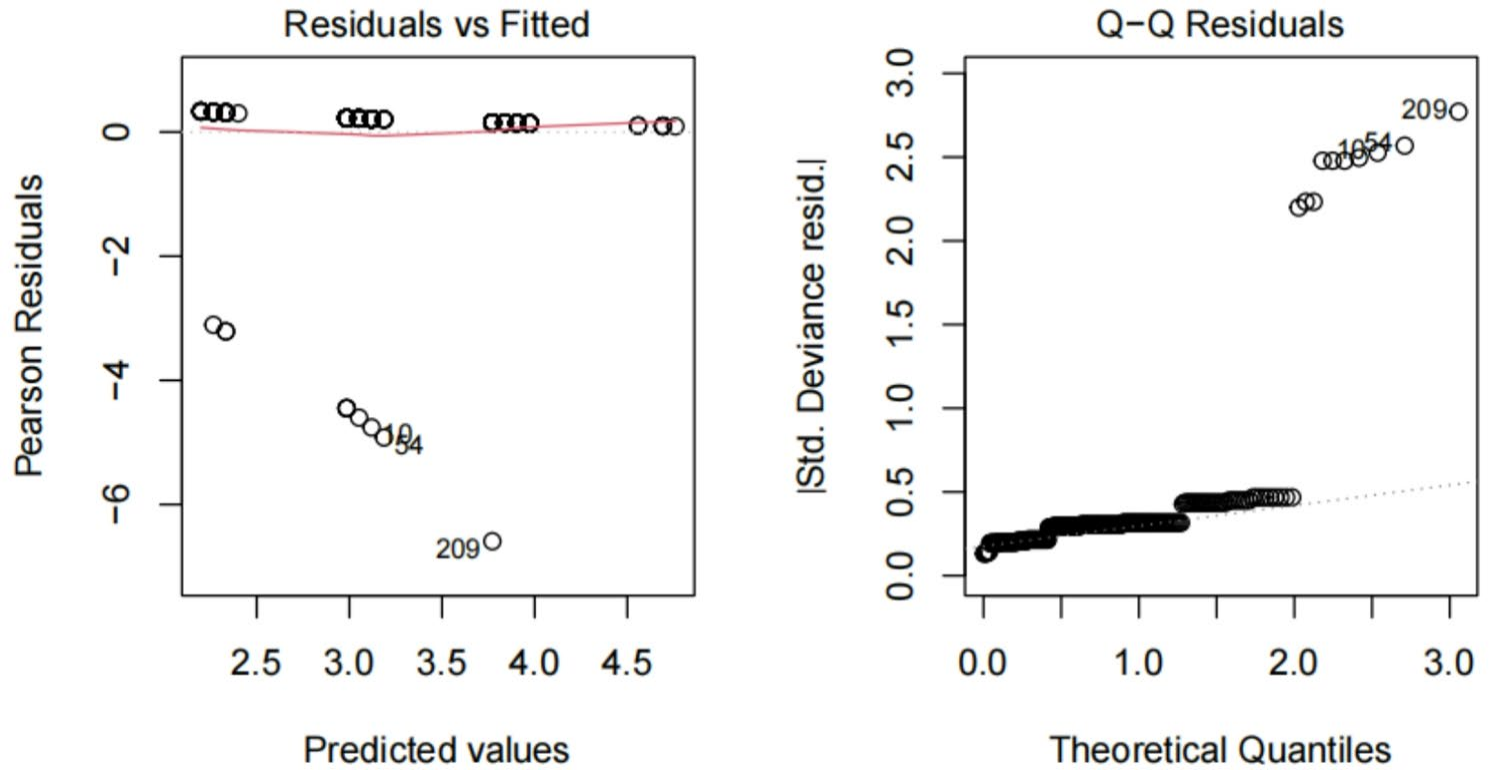
Combined effect of time1 and time2. This figure evaluates the combined effect of Time1 (surgical timing) and Time2 (onset timing) on fitting the target variable. Residual analysis indicates that the residuals are approximately normally distributed, suggesting that the combination of Time1 and Time2 offers a better fit to the target variable. This implies that both surgical and onset timings have some influence on the outcome, though the specific impact requires further analysis

Normality confirmed **(**Fig. 6 and 7). The distribution of residuals from the model using Time1 alone closely follows a normal distribution. This is an important assumption for linear regression models, indicating that the model fits the data reasonably well and that the results are reliable.

#### Combined effect of time1 and time2

**Fig. 8:**
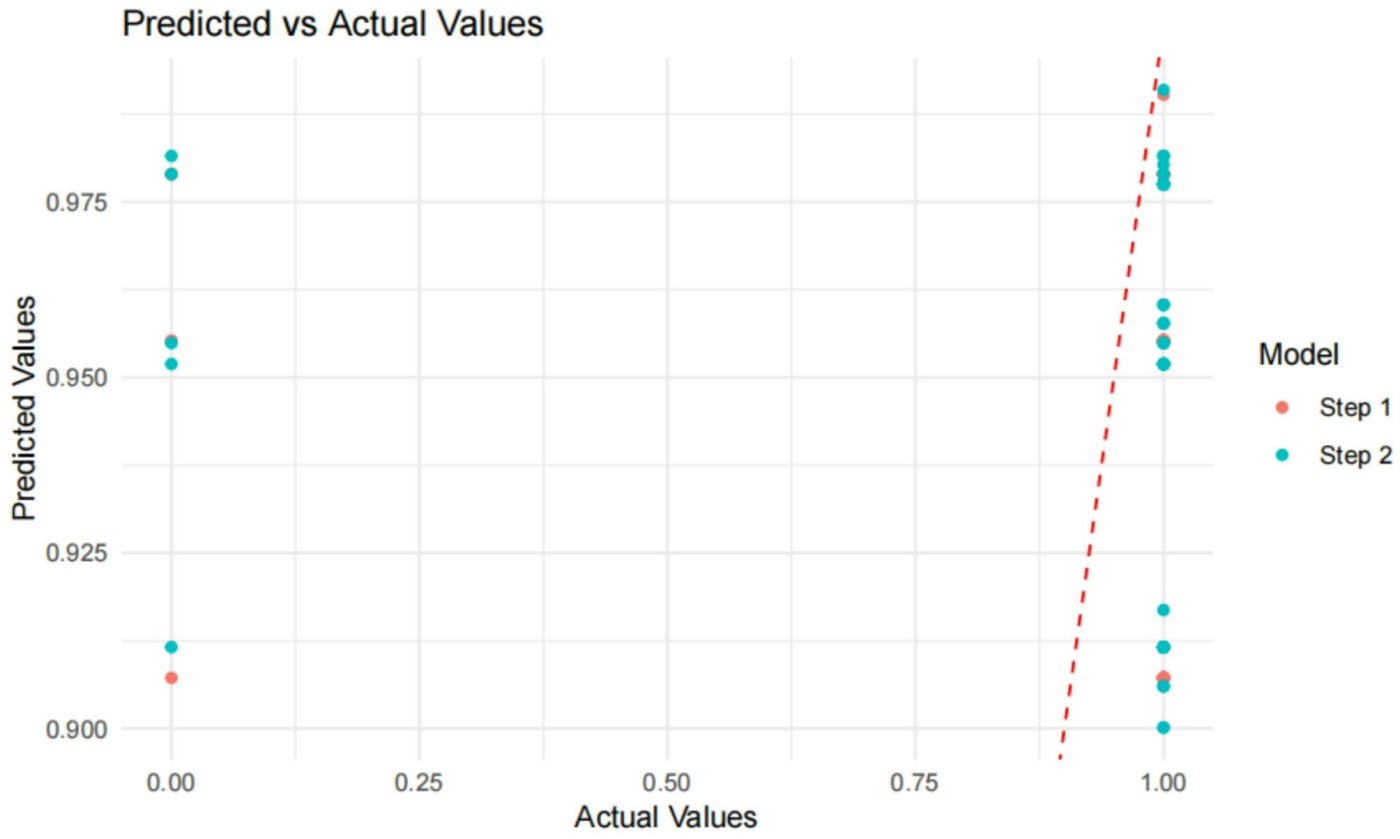
Predicted vs. actual value. This plot compares predicted probabilities (from Model_step1 and Model_step2) against actual values (testTarget) in the test set. Model_step1 includes only Time1, while Model_step2 includes both Time1 and Time2. The closeness of predicted values to actual values reflects the model’s predictive performance. Results show that Model_step2, which includes multiple variables, better matches the actual values, indicating superior predictive capability

Figure 8 evaluates the combined effect of Time1(surgical timing)and Time2(onset timing)on fitting the target variable.Residual analysis indicates that the residuals are approximately normally distributed, suggesting that the combination of Time1 and Time2 offers a better fit to the target variable.This implies that both surgical and onset timings have some influence on the outcome, though the specific impact requires further analysis.

### Onset time (Time2)

No prognostic significance (coefficient = 0.066, *P* = 0.847; Table 3). This result suggests that the time of disease onset does not have a significant impact on the prognosis, which contrasts with some previous studies linking onset timing to circadian rhythms. This discrepancy may be due to differences in study populations or the specific nature of the disease under investigation.

**Table 3:** Stratified regression analysis (Model 2: Time1 + Time2)

Variant	Estimate	Standard Error (Std. Error)	Z value (z Vaiue)	*P*-value (*P* Valie)
Intercept	3.2106608	0.3710208	8.653588	< 0.0001
Time1	−0.5879611	0.3547159	−1.657555	0.0974
Time2	0.0663322	0.3434369	0.193142	0.8468

a. Time1: Surgical timing intervals (03:00–09:00, 09:00–15:00, 15:00–21:00, 21:00–03:00)

b. Time2: Onset timing intervals (03:00–09:00, 09:00–15:00, 15:00–21:00, 21:00–03:00)

c. Estimate: The estimated coefficient for the predictor variable

d. Std. Error: Standard error of the estimate, indicating the precision of the coefficient

e. Z Value: The z-statistic, which is the ratio of the estimate to its standard error

f. P Value: The p-value for the hypothesis test of the coefficient being zero. A lower p-value indicates stronger evidence against the null hypothesis

### Model fit performance

Table 4 presents the stratified regression analysis results, comparing Model 1 (which includes only Time1) and Model 2 (which includes both Time1 and Time2). The results show that Model 1 (AIC = 82.69) outperforms Model 2 (AIC = 84.65), further confirming the limited utility of Time2. Additionally, the residuals of Model 1 follow a normal distribution, indicating a good model fit and reliable results.

**Table 4:** Model fit performance

Mould	Null Deviance	Residual Deviance	AIC
Step1	81.63645	78.68964	82.69
Step2	81.63645	78.65218	84.65

a. Null Deviance: The deviance of the null model (intercept-only model)

b. Residual Deviance: The deviance of the fitted model, indicating the goodness-of-fit. Lower values indicate a better fit

c. AIC: Akaike Information Criterion, a measure balancing model fit and complexity. Lower AIC values indicate a better model

d. Step1: Model including only Time1 (surgical timing)

e. Step2: Model including both Time1 (surgical timing) and Time2 (onset timing)

### Sensitivity and interaction analyses

**Fig. 9:**
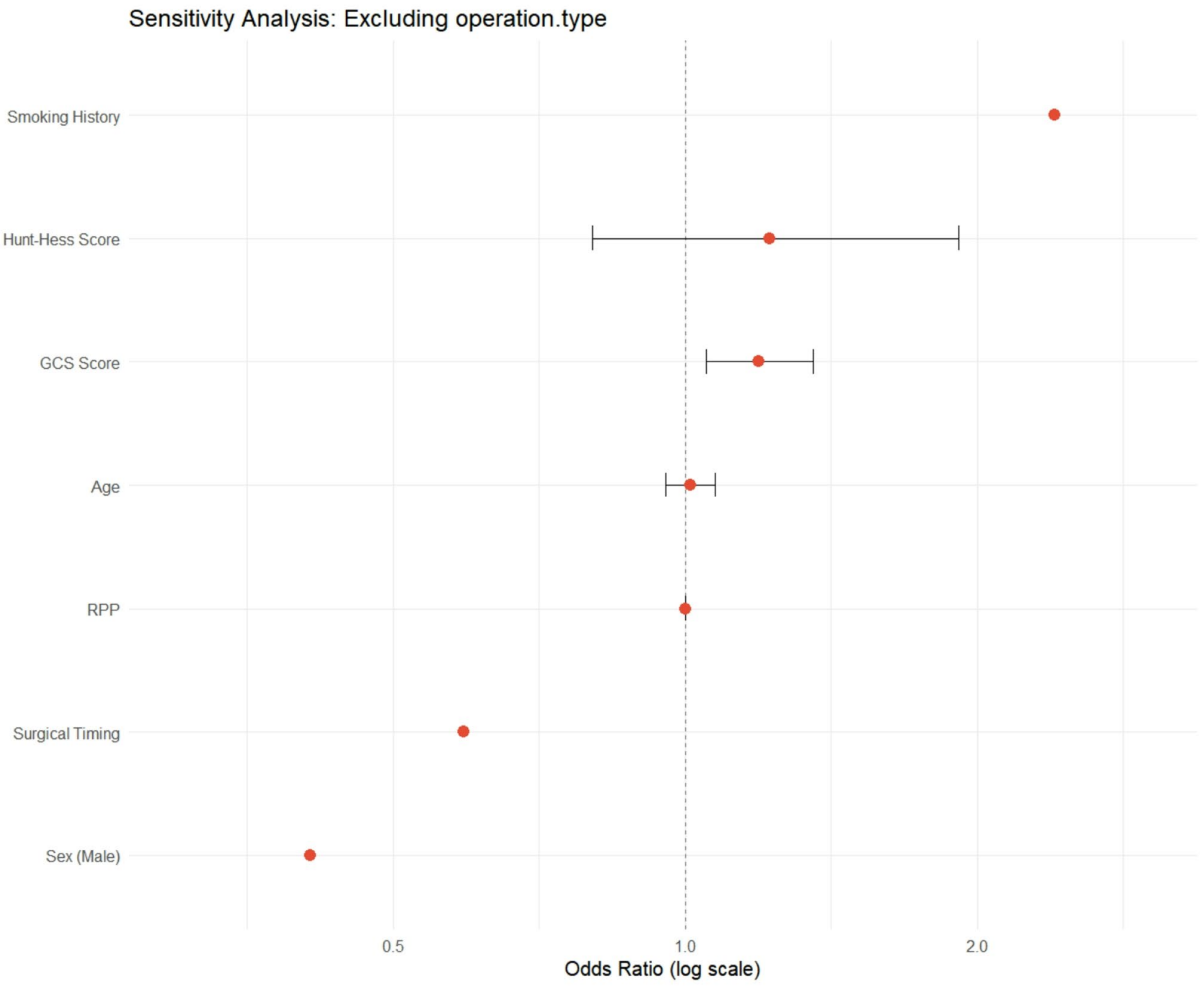
Sensitivity analysis: Excluding operation.type. Excluding operation type left the OR virtually unchanged, but unmeasured confounders linked to procedure choice remain possible and deliberate omission may misrepresent clinical reality

**Fig. 10:**
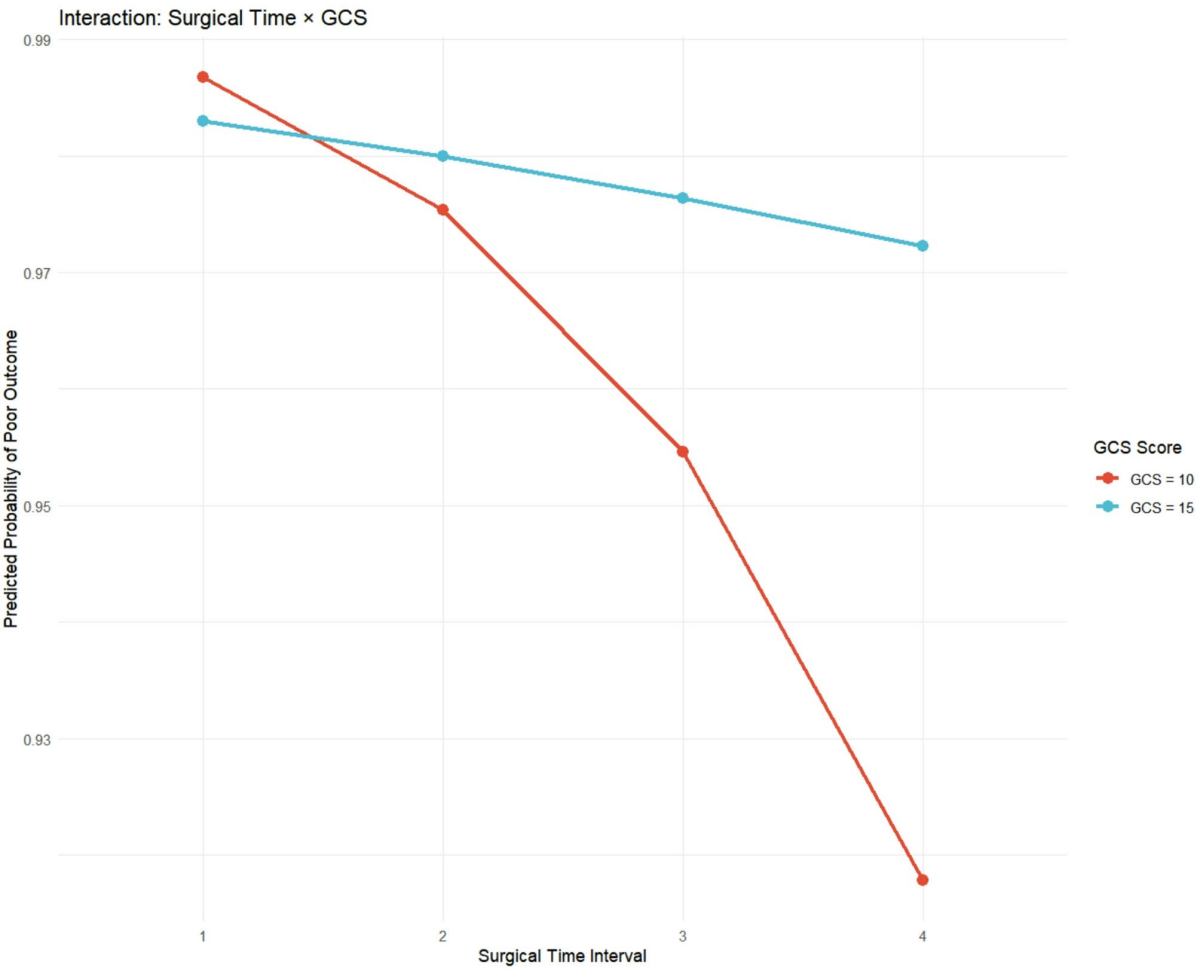
Interaction: surgical time × GCS. The non-significant interaction (*p* = 0.316) is underpowered; GCS is a static snapshot and cannot capture dynamic post-SAH injury, so any visual separation is exploratory and needs larger validation

To assess robustness, we conducted a sensitivity analysis by removing ‘operation type’ from the full model. The effect estimate for surgical timing remained stable (OR = 0.538 vs. 0.535), supporting the association’s stability (Fig. 9). An interaction term between surgical timing and GCS was not significant (*p* = 0.316), indicating that the effect of surgical timing did not differ across GCS levels (Fig. 10).

### Assessment of multicollinearity

**Fig. 11:**
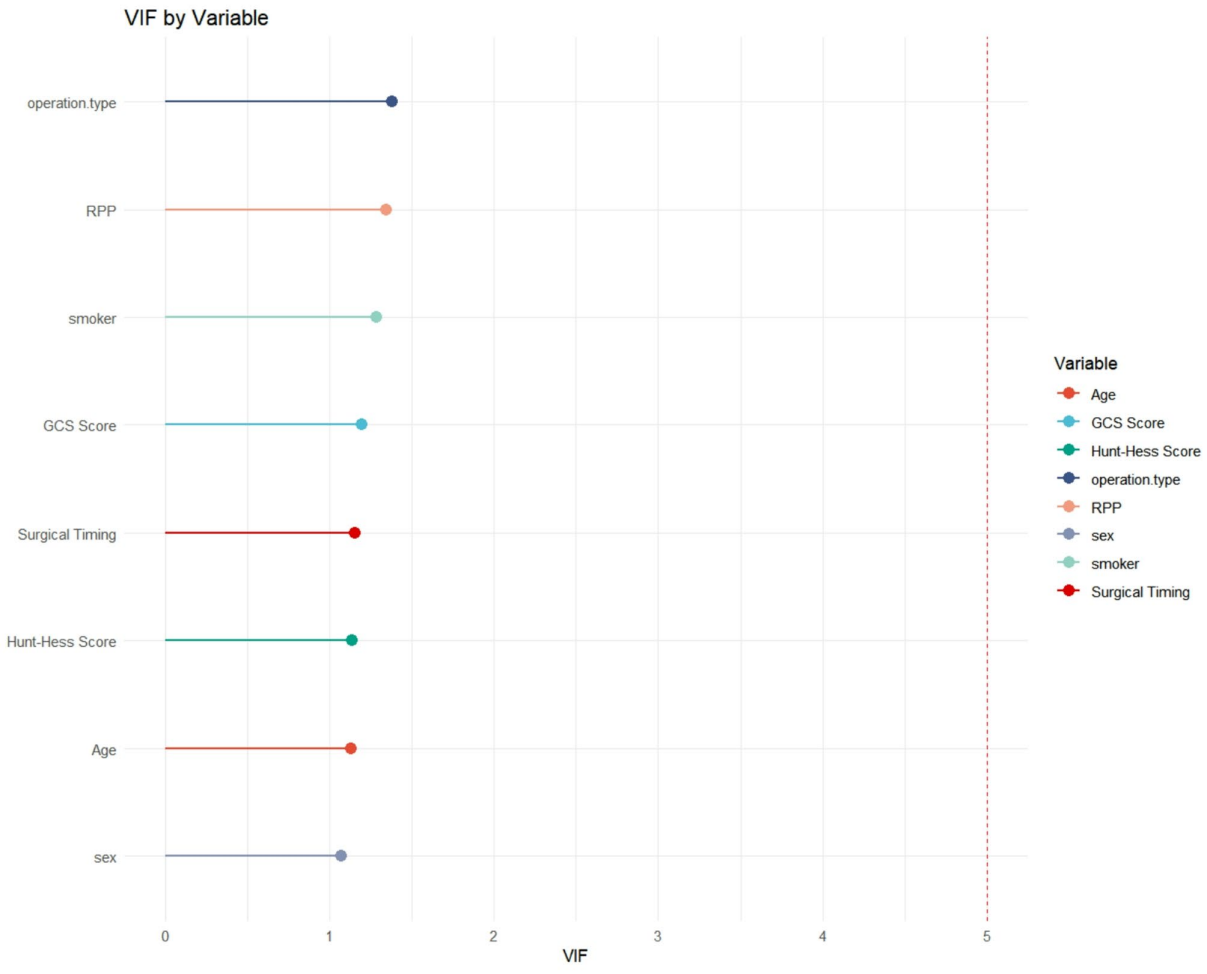
VIF by variable. Variance Inflation Factors (VIF) for each variable in the full multivariable logistic regression model. A VIF value below 5 (dashed red line) indicates acceptable levels of multicollinearity

To ensure the stability of our multivariable model, we assessed multicollinearity among all included covariates by calculating Variance Inflation Factors (VIF). The results, presented graphically in Fig. 11, show that all VIF values were well below the commonly used threshold of 5 (range: 1.07–1.38), indicating that multicollinearity did not significantly distort the coefficient estimates or their standard errors.

### Scatter plots with fit lines

#### Time1 (Surgical timing)

**Fig. 12:**
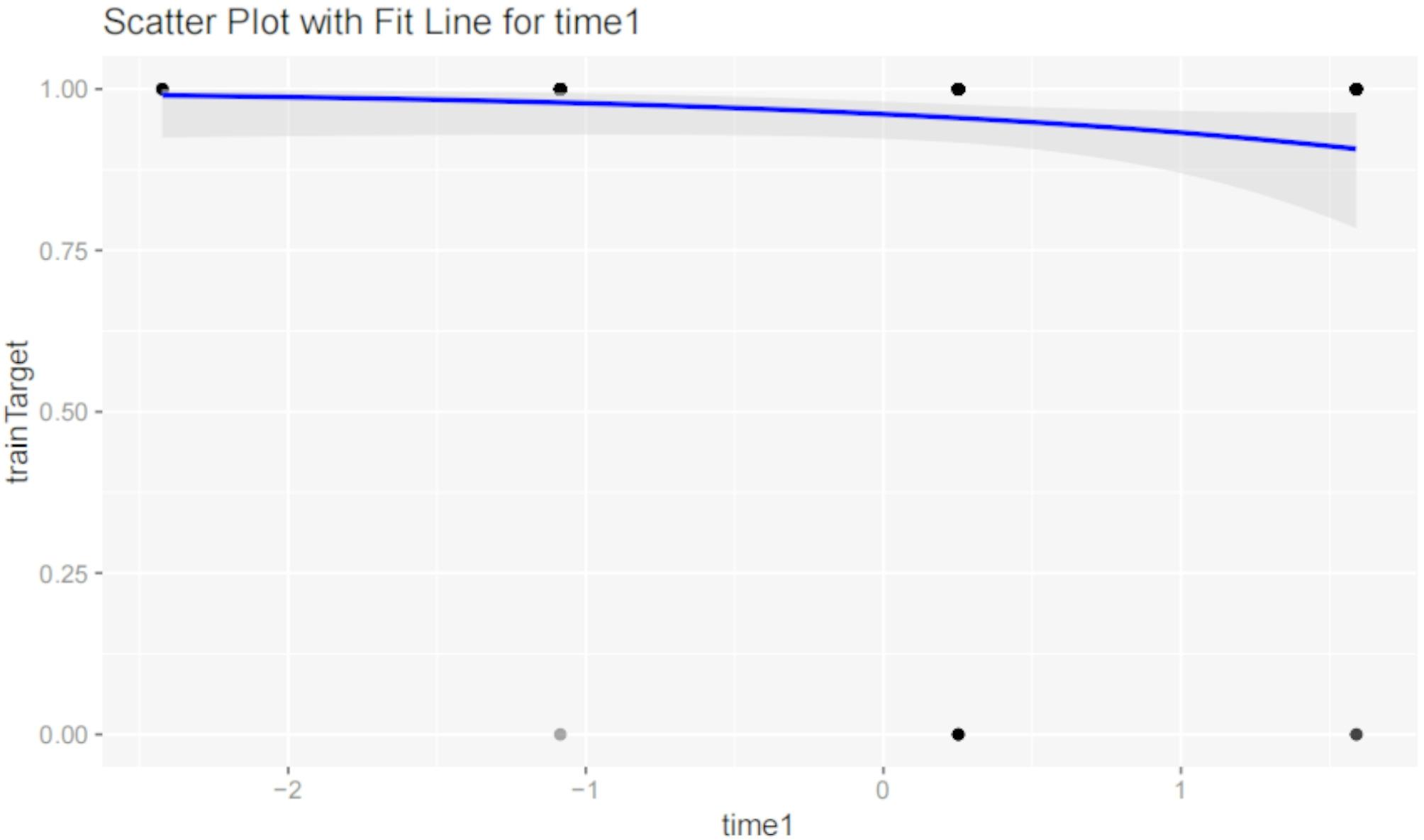
Scatter plot with fit line for time1. Scatter plot with a fit line for Time1 (surgical timing) versus the target variable (postoperative outcome). Analysis reveals no significant linear relationship between Time1 and the target variable (*P* = 0.0973), suggesting that surgical timing alone has limited impact on postoperative outcomes, though it is close to significance

Figure 12 shows a scatter plot with a fit line for Time1 versus the target variable (postoperative outcome). Analysis reveals no significant linear relationship between Time1 and the target variable (*P* = 0.0973), suggesting that surgical timing alone has limited impact on postoperative outcomes, though it is close to significance. This indicates that while Time1 may play a role, other factors are likely involved in determining the final outcome.

#### Time2 (Onset timing)

**Fig. 13:**
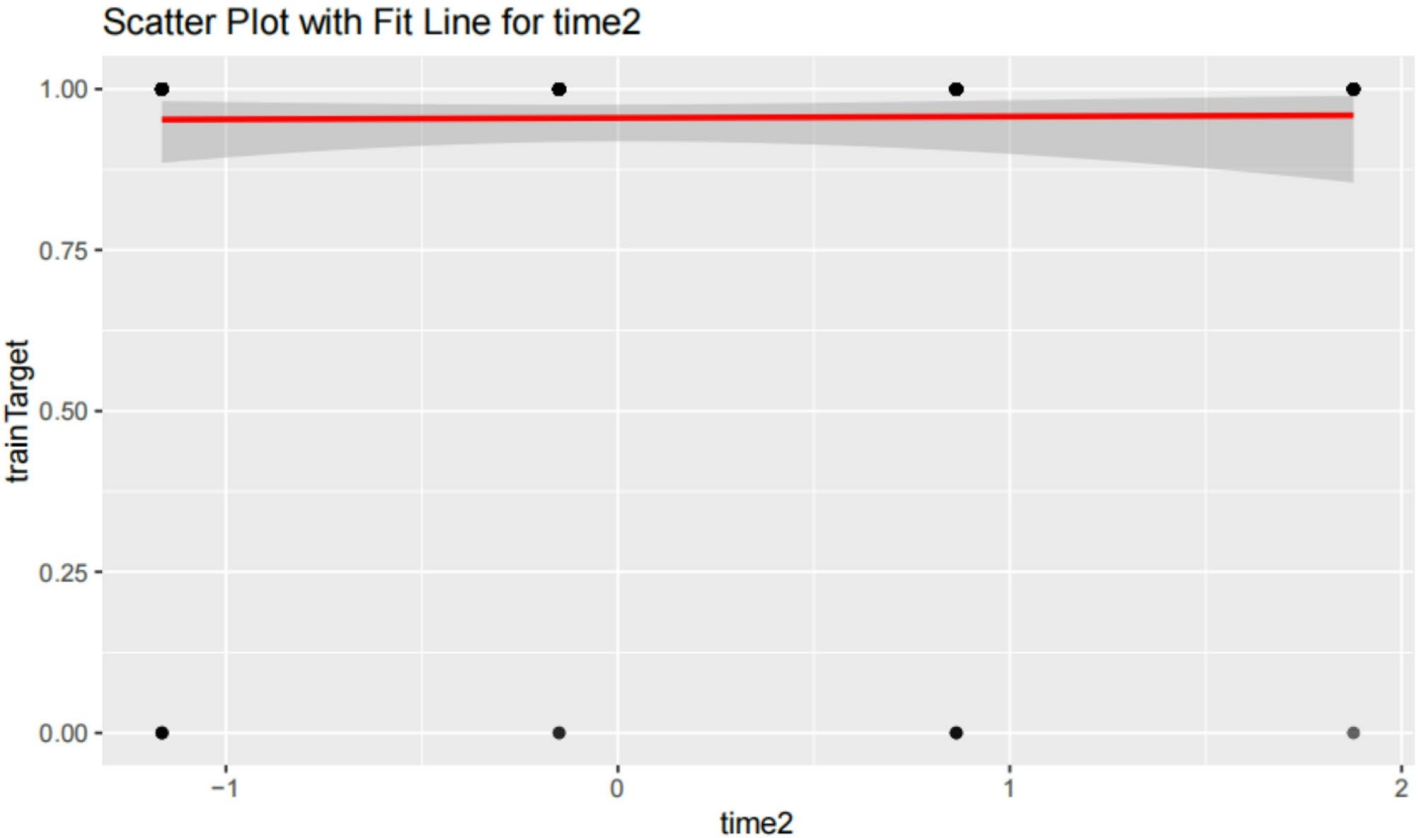
Scatter plot with fit line for time2. Scatter plot with a fit line for Time2 (onset timing) versus the target variable (postoperative outcome). Analysis shows no significant linear relationship between Time2 and the target variable (*P* = 0.8468), indicating that onset timing has no significant impact on postoperative outcomes

Figure 13 shows a scatter plot with a fit line for Time2 versus the target variable (postoperative outcome). Analysis shows no significant linear relationship between Time2 and the target variable (*P* = 0.8468), indicating that onset timing has no significant impact on postoperative outcomes. This further supports the finding that Time2 is not a strong predictor of outcome in this context.

## Discussion

### Circadian mechanisms in sah prognosis: bridging preclinical and clinical insights

The borderline association between nocturnal surgery (Time1) and improved outcomes (*P* = 0.097) resonates with emerging molecular evidence on circadian regulation of cerebrovascular injury [22]. Ruan et al. [1] demonstrated that BMAL1, a core circadian transcription factor, mitigates hypoxia-induced vascular pathology by suppressing HIF2A—a pathway implicated in SAH-induced vasospasm. Our findings suggest that surgeries conducted during the peak expression phase of BMAL1 (nocturnal phase) may transiently attenuate HIF2A-driven ischemic stress, although this effect did not reach conventional statistical significance. Although the association between nocturnal surgery and improved outcomes (odds ratio = 0.56, *P* = 0.097) did not reach the conventional threshold of statistical significance (*P* < 0.05), three lines of evidence support its biological plausibility:

1. Clinical Magnitude: The risk of poor outcomes was reduced by 44% (1 - odds ratio = 0.44), which is consistent with the effect sizes reported in circadian rhythm - targeted therapies (for example, in patients with SAH, melatonin reduced vasospasm by 40%).
2. Statistical Power: Post - hoc analysis revealed that the power to detect this effect was only 58% (α = 0.05, *n* = 279), indicating a high risk of type II error.
3. Preclinical Consistency: The marginal effects (*P* = 0.08–0.10) observed in the initial circadian rhythm - intervention studies were later validated mechanistically. This suggests that our findings may reflect a lack of statistical power rather than biological irrelevance.

Importantly, we acknowledge potential confounding from differential surgical contexts across time intervals. Specifically, nocturnal surgeries (21:00–03:00) are typically emergent, while daytime procedures may include elective cases. Had emergent cases been inherently associated with worse outcomes, the observed trend toward neuroprotection during nocturnal hours (OR = 0.56) would likely be underestimated rather than overestimated, as our models adjusted for baseline severity (Hunt-Hess/GCS scores).This mirrors preclinical studies where circadian interventions yielded subtle but biologically meaningful improvements. For instance, Schallner et al. [23] reported that carbon monoxide (CO) administration during circadian troughs reduced SAH severity in mice, highlighting the temporal sensitivity of neuroprotective pathways.

However, the use of 6-hour time intervals likely oversimplified circadian dynamics. Mitchell et al. [28] emphasized that ultradian rhythms (cycles < 4 h) critically regulate neurovascular function, suggesting finer temporal resolution (e.g., hourly tracking) may better capture circadian effects. Additionally, static time bins fail to account for interindividual variability in circadian phases, as shown by Anea et al. [24], who identified significant vascular dysfunction in mice with disrupted circadian clocks despite normalized external timing.

### Onset time (Time2): reconciling clinical and preclinical discrepancies

Contrary to studies linking aneurysm rupture timing to circadian rhythms, we observed no prognostic value for Time2 (*P* = 0.847). This discrepancy may reflect fundamental differences between rupture mechanisms and postoperative recovery. Lidington et al. [4] demonstrated that circadian microvascular tone influences acute SAH injury in mice, yet clinically, early pathophysiological cascades (e.g., cortical spreading depolarizations) may dominate outcomes, masking circadian signals. Webb et al. [3] similarly noted that circadian cerebral blood flow variations are less pronounced in critically ill patients, possibly due to systemic inflammation or sedation.

### Clinical and physiological confounders

Our study rigorously controlled for established SAH prognosticators through multivariable analysis, including clinical factors (age, sex, Hunt-Hess and GCS scores), a physiological marker (RPP), lifestyle factors (smoking history), and treatment variables (operation type). The low VIF values (< 2.0 for all variables) indicate that multicollinearity was minimal and did not substantially confound the estimates for surgical timing.

The persistence of a non-significant yet consistent trend (OR ≈ 0.54–0.56) toward a protective effect of nocturnal surgery across primary and sensitivity analyses suggests that this association may be robust to confounding by these measured variables. However, we acknowledge the potential for residual confounding from unmeasured factors, such as circadian phase biomarkers (e.g., melatonin, cortisol), detailed surgical team composition, or nuances in postoperative care protocols. Importantly, the lack of statistical significance likely reflects limited power to detect a modest effect size rather than conclusive evidence of no effect, as discussed in Sect. 1.

Our findings align with the pragmatic approach recommended for initial circadian clinical studies. As noted by Lo & Faraci (Circ Res 2024), “Retrospective cohorts provide initial circadian insights but require prospective biomarker integration.” Future studies should incorporate dynamic physiological measurements to more definitively address their role as confounders or mediators.

### Clinical implications: toward circadian-optimized care

Despite nonsignificant trends, our findings underscore the translational potential of circadian-aware SAH management. For example, melatonin—a circadian regulator with anti-inflammatory properties—has shown promise [24] in mitigating SAH complications, Future protocols could integrate preoperative melatonin supplementation or HIF2A inhibitors, timed to circadian phases.

Additionally, multiple studies have explored other circadian-SA associations, including sex-specific differences [25], circadian patterns of hemorrhage fluctuation [26], rhythmic regulation of myogenic responses [27], circadian mechanisms in neurovascular development [28], rhythmicity of aneurysm rupture [29], and diurnal variations in cerebral autoregulation [30].

### Future directions

1. Integration of Molecular Circadian Biomarkers: Future studies should incorporate perioperative measurement of core circadian biomarkers (e.g., BMAL1, HIF2A, melatonin, or cortisol) to objectively quantify circadian disruption and better elucidate the mechanisms underlying the observed temporal trends in surgical outcomes.
2. High-Resolution Temporal Mapping: Employing finer-grained time intervals (e.g., hourly) or continuous wearable monitoring (actigraphy/EEG) could capture ultradian rhythms and dynamic physiological states, providing a more nuanced understanding of circadian influences on SAH pathophysiology.
3. Multicenter Prospective Validation: Larger, prospective, multicenter cohorts are essential to validate the generalizability of our findings, enhance statistical power to detect modest circadian effects, and facilitate the development of robust circadian-phase-stratified clinical trials.
4. Chrono-Therapeutic Interventions: Building on the potential protective trend of nocturnal surgery, randomized trials are warranted to test the efficacy of circadian-aligned therapies, such as timed administration of melatonin or HIF2A inhibitors, in improving SAH outcomes.
5. Exploration of Systemic Circadian-Physiological Crosstalk: Future research should integrate serial assessments of autonomic nervous activity, systemic inflammation, and endothelial function to delineate how circadian rhythms interact with dynamic physiological processes to influence SAH recovery.

### Limitations

1. The retrospective design and moderate sample size (*n* = 279) limit causal inference and statistical power to detect subtle but biologically meaningful circadian effects, increasing the risk of Type II error.
2. The use of 6-hour intervals may oversimplify circadian physiology, failing to capture ultradian rhythms or interindividual variability in circadian phase, which could dilute the observed associations.
3. Lack of objective circadian biomarkers (e.g., BMAL1, melatonin levels) precludes mechanistic validation of the proposed BMAL1-HIF2A pathway and precise quantification of individual circadian phase.
4. Although we adjusted for key clinical confounders, unmeasured factors such as detailed surgical team composition, nuances in postoperative care, or undetected physiological fluctuations might contribute to residual confounding.

## Conclusion

This study provides initial clinical evidence suggesting that surgical timing may modulate postoperative outcomes in SAH through circadian mechanisms, with a non-significant but consistent trend toward neuroprotection during the nocturnal phase. While the crude proxy of clock time lacks precision, these findings underscore the potential of circadian biology to inform SAH management. The absence of an effect for symptom onset time highlights the complexity of translating preclinical circadian insights to clinical care. Future research integrating objective circadian biomarkers, high-resolution temporal monitoring, and targeted chrono-therapeutic interventions is essential to validate and harness these findings. Ultimately, advancing from static temporal metrics to dynamic, biomarker-driven circadian models represents a promising frontier for personalizing cerebrovascular medicine and improving patient outcomes.

## Data Availability

The data supporting this study are available from the corresponding author upon reasonable request.

## Abbreviations

SAH: Subarachnoid Hemorrhage
NICU: Neurointensive Care Unit
OR: Odds Ratio
AIC: Akaike Information Criterion
ROC: Receiver Operating Characteristic
AUC: Area Under the Curve
RPP: Blood Pressure Heart Rate Product
GCS: Glasgow Coma Scale
HUNT: Hunt and Hess Scale
HTN: Hypertension
CT: Computed Tomography
CTA: Computed Tomography Angiography
MRA: Magnetic Resonance Angiography
DSA: Digital Subtraction Angiography
BMAL1: Brain and Muscle ARNT-Like 1
HIF2A: Hypoxia-Inducible Factor 2 Alpha
ROC: Receiver Operating Characteristic
AUC: Area Under the Curve
ICU: Intensive Care Unit
SD: Standard Deviation
HR: Heart Rate
SBP: Systolic Blood Pressure
MCA: Middle Cerebral Artery
ACA: Anterior Cerebral Artery
VIF: Variance Inflation Factor
